# Seasonal changes and variability of physical match demands in a highly trained female soccer team

**DOI:** 10.5114/biolsport.2026.156226

**Published:** 2025-11-21

**Authors:** João Barreira, Pedro Figueiredo, Cristian Petri, Luca Pengue, Francesco Perondi, Alessandro Buccolini, Jaime Fernandez-Fernandez, Fábio Y. Nakamura

**Affiliations:** 1 Research Center in Sports Sciences, Health Sciences and Human Development, CIDESD, University of Maia, ISMAI, Maia, Portugal; 2 Physical Education Department, College of Education, United Arab Emirates University, Al Ain, United Arab Emirates; 3 Department of Sport and Informatics, Section of Physical Education and Sport, Pablo de Olavide University, 41013 Sevilla, Spain; 4 A.C.F. Fiorentina S.r.l., Florence, Italy; 5 Department of Physical Education and Sport Sciences, Universidad de León, León, Spain; 6 AMRED, Human Movement and Sports Performance Analysis, Universidad de León, León, Spain; 7 Graduate Program in Physical Education, Federal University of Pernambuco, Recife, Brazil

**Keywords:** Performance analysis, High-speed running, Women, Football, Match performance

## Abstract

This work aimed to identify the sources of variability in match physical performance of highly trained female soccer players and quantify individual between-match changes in global positioning systems (GPS) derived metrics. Official match data belonging to 27 players across two competitive seasons was analyzed, resulting in 344 individual observations. GPS metrics included total distance covered (TD), distance covered per min, high-speed running distance covered (HSRD, 19.8–25.2 km/h), high-metabolic load distance (HMLD, > 20 W/kg), maximal speed (Max speed (km/h)), and high-intensity accelerations and decelerations distances covered (> 2.5 m/s^−2^). Seasonal variation trends and variability across players, positions, and matches were quantified using linear mixed models. Reference values for interpreting between-match changes were established based on observed match-to-match variability and the smallest worthwhile changes. Results indicated that lower-intensity metrics such as TD, m/min, and max speed were relatively stable, with a minimum detectable change of approximately ± 14%. In contrast, high-intensity metrics such as HSRD, HMLD, and acceleration/deceleration distances demonstrated greater variability, requiring changes > 30% to exceed expected fluctuations. Considering the cohort of the study, findings suggest that high-intensity metrics present the highest variability and, consequently, considerably higher minimum detectable change thresholds. Practitioners may consider the variability thresholds presented when interpreting match-to-match changes and individualizing player monitoring strategies.

## INTRODUCTION

Soccer match-play physical demands have been attracting attention from several research groups over the past years. Numerous studies involving players from different countries and playing levels, using different tracking systems, have been published and summarized in reviews [1–3]. Although some reports involve full-season training and match data, the main aim has been to determine the training load during congested and regular microcycles [4, 5] and weekly ‘periodization’ models [6, 7] centered on the match day. Less is known about match performance over an entire season to determine whether competitive physical demands are maintained or changed over time. This becomes increasingly important with the rise in physical demands of modern soccer competitive seasons, where teams often play two matches within a single week [8].

Thus, assessing match performance is fundamental to developing subsequent training and recovery programs and improving performance [9]. However, match performance is a multifactorial construct with a dynamic and stochastic nature, where a player’s physical performance is affected by multiple external factors (i.e., ball possession, opponent level, and weather, and player status) [10]. While much of the existing literature has focused on male cohorts, it is increasingly clear that female soccer possesses unique physical, tactical, and contextual characteristics [11]. Differences in match tempo, sprint frequency, match congestion [12], and hormonal considerations [13] can all influence physical performance and variability patterns in the women’s game. For instance, recent evidence has demonstrated substantial inter-positional and contextual effects on match loads in elite female players, suggesting that findings from male populations cannot be readily generalized. A review by Pérez Armendáriz et al. [12] highlighted key sex-specific considerations in match demands across female team sports, reinforcing the need for tailored monitoring and training approaches in women’s football. In addition, other factors such as coaching staff [14], weather conditions [15], substitutions [16], and physical fitness [17] might also modulate activity patterns of a game. This means that multiple contextual factors might be associated with match-to-match locomotor performance variability in soccer players. Such variability precludes clearly observing shortterm ascending or descending activity patterns over consecutive matches; however, looking at the whole season might help identify patterns despite the inter-match activity variability.

Therefore, quantifying the match-to-match variability of physical performance can be a useful strategy to compute changes that may seem typical and those that might be higher or lower than usual. One method proposed is the coefficient of variation (CV). It seems that high-intensity running is the most inconsistent/highly variable metric across the matches [9, 18]. With a large sample, Gregson et al., [10] reported that the high-speed activity parameter showed a coefficient of variation of 15.1 to 32.6% in male players over three Premier League seasons. The authors concluded that this high match-to-match variability in the high-speed activity precludes using the associated metrics to quantify an individual’s fitness capacity. However, using this quantitative approach in isolation does not quantify the magnitude of a change. More recently, scaling changes against reference values for practical importance (i.e., smallest worthwhile change [SWC]) have been suggested. Then, probabilistic methods can be used to determine the ‘practical significance’ of individual changes, considering both match-to-match variability and the SWC [19, 20]. For instance, McLaren et al. [19] estimated the minimum threshold required for a substantial within-player (and between-match) change in match physical performance to be interpreted as ‘likely’ (75% chance) in rugby union players. For example, regarding high-intensity activities, the authors reported that the change required to likely be significant is considerably high (21–76%), meaning that an increase/decrease of 21-76% would be necessary, which is a result of the high variability in this type of variables.

Following this approach, Baptista et al. [21] and Oliva-Lozano et al. [22] analyzed the match performance variability and identified a minimum detectable change in male and female soccer players, respectively. Baptista et al. [21] analyzed the match-to-match variability of four elite female soccer teams over a one-season period, reinforcing that different sources of variability (i.e., between-match, player, and team) seem to affect players’ match physical performance. However, this is the only study with female adult soccer players. In female academy soccer players, Myhill et al. [23] found high within-player and between-match variability in acceleration and high-speed running metrics among female academy players. Accordingly, and in line with these studies, our work’s aims were threefold: 1) to describe and compare the match external loads of a professional women’s soccer team between two competitive seasons and within each season; 2) to analyze and provide a comprehensive breakdown of the variability of each external load metric, including seasonal trends, across two seasons, and 3) to identify the minimum individual detectable changes in female soccer match physical performance indicators.

## MATERIALS AND METHODS

### Study design

This observational study was conducted in a professional soccer female team that participated in the Serie A Women League and *Coppa Italia* Women during the 2021/2022 and the 2022/2023 competitive seasons. The team played a total of 55 official matches (season 1 [21/22] = 25; season 2 [22/23] = 30). There were 28 away matches and 27 matches played at home. Each season was 39 weeks long. Data arose from the regular monitoring of match loads regularly performed by the club staff. The study was approved by the Ethics Committee of the University of Maia (210/2024).

### Subjects

Data from 27 highly trained female soccer players who were monitored throughout a 2-season period are reported in this study. Fifteen players were monitored during the 21/22 season, and 19 players were observed during the 22/23 season, with 7 players participating in both seasons, resulting in 432 individual match observations. Goalkeepers were not included in the analysis and only data from players who completed the full 90 minutes of the match were retained for the analysis of match-to-match physical performance variability. This exclusion criterion reduced the final sample to 344 individual match observations, of which 196 belonged to players who participated in both seasons. Players were further categorized into 5 different playing positions: wide defenders (n = 69 [individual observations]), center backs (n = 87), midfielders (n = 112), wingers (n = 18), and forwards (n = 58). Playing position was adjusted to the role the player had on each specific match (in the starting XI), meaning that a given player may have played more than one role throughout the season(s) (e.g., played as a midfielder on matches 2, 3, 4, and then as a center-back on match 5 and 6). According to the participants’ classification framework, players in the current study were classified as Highly-trained/National level [24].

### External Match loads

Players wore an 18-Hz Global Positioning System (GPS) (GPEXE Pro 2, GPEXE, Udine, Italy) [25] during all official matches analyzed throughout the two seasons. External load metrics included total distance covered (TD), distance covered per min (m/min), high-speed running distance covered (HSRD, 19.8-25.2 km/h), high metabolic load distance (HMLD, distance covered with power consumption above 20.0 W/kg), maximal speed (Max speed (km/h)), and highintensity accelerations and decelerations distances covered (> 2.5 m/s^−2^). These thresholds were defined by the club’s staff without any interference from the research team. It is also worth noting that intensity thresholds considerably vary across studies [3]. All metrics were expressed as absolute values for full 90-minute matches. No time-normalization or adjustment for effective playing time was applied.

### Statistical Analysis

Data were analyzed in the R software (“Spotted Wakerobin” Release for macOS, R Core Team, 2023). After importing the dataset into the software, an exploratory data analysis was conducted, and assumptions of normality and heterogeneity were verified. Descriptive statistics (mean ± SD) were used to characterize the external workload profile metrics. Linear mixed models were employed because the clustered dataset is characterized by repeated measures from the same subject across numerous time points. All models were estimated via Restricted Estimated Maximum Likelihood (REML), and model appropriateness was verified by examining the QQ-plots of the studentized residuals. Each random effect represented a source of variability and was expressed in raw units (standard deviation, SD) by modelling the original data, also expressed in percentage units (CV%), by first log-transforming the original data before modelling, and then back-transforming each estimate after modelling was done [26]. For this, the dependent variable was first transformed (100*ln(y)) and then back-transformed using the formula 100*(exp(x/100)-1). Ninety percent confidence intervals (CI) of the variance components were obtained by bootstrap.

To compare the external match workloads between seasons, external load metrics were considered the dependent variable, and the competitive season was a fixed effect. Player ID, match ID, and player position were considered random effects. To assess the linearized seasonal trend in each external load metric, the seasons were analyzed separately. Again, external load metrics were considered the dependent variable, and the re-scaled (-0.5 to 0.5) season week was specified as a fixed effect (continuous covariate). Player ID, match ID, and player position were considered random effects.

Following the methodology used by Oliva-Lozano et al. [22] and Baptista et al. [21], variability estimates were used to provide a framework for practitioners to interpret individual changes in external match load metrics. The objective here is to identify changes that appear unusual and potentially meaningful. This unusual change can be interpreted as one beyond the normal match-to-match variability seen in any given player, after considering any seasonal trend and positional difference. The observed match-to-match variability was determined as the pooled (added) between-match SD and within-player typical error. These values were then multiplied by the square root of 2 with the appropriate values from the t-distribution (with infinite degrees of freedom) to establish 80% and 90% CI, giving likely ranges for a normal or ‘usual’ individual change. Furthermore, practically significant changes associated with ‘conventional’ alpha levels of 0.10 and 0.05 were calculated using the formula: * observed between-match variability * t-statistic + threshold. Here, the observed between-match variability was the same as described above, while the threshold term was equivalent to the smallest worthwhile change (0.2 * the observed between-player variability or the pooled betweenplayer and within-player variability).

## RESULTS

External match loads for each season as well as mean values of both seasons are presented in Table 1. Season 2 presented significantly higher HSRD during the matches than season 1 (+36.9 m, p = 0.02, d = 1.09 [95% CI = 0.04; 2.12]). There were no statistically significant differences between seasons on the remaining external load metrics.

**TABLE 1:** Descriptive statistics of the match external loads of each season and the mean of the two seasons.

Metric	Mean	Seasons		Model	

		Season 1	Season 2	F	p
Total Distance (m)	9290.6 ± 720.3	9219.4 ± 664.0	9357.5 ± 765.4	2.93	0.09
m/min (m)	103.2 ± 8.0	102.4 ± 7.4	104.0 ± 8.5	2.93	0.09
HSRD (m)	288.6 ± 123.4	266.6 ± 113.4	309.2 ± 129.2	4.91	0.02
HMLD (m)	1982.3 ± 366.4	1962.5 ± 297.5	2000.9 ± 421.0	3.12	0.08
Max Speed (km/h)	26.3 ± 1.7	26.3 ± 1.8	26.4 ± 1.7	0.07	0.79
Accelerations (m)	40.6 ± 15.0	41.3 ± 15.2	40.1 ± 14.9	1.19	0.27
Decelerations (m)	81.3 ± 25.1	85.1 ± 26.3	77.7 ± 23.4	0.03	0.84

HSRD = high-speed running distance; HMLD = high metabolic load distance.

### External match loads variability

The decomposed variability of the external match load analysis is presented in Table 2. All estimates of between-match, between-season, between-position, between-player, and within-player variability are expressed in raw (SD) and percentage (CV%) units. The CV values of the external match loads ranged from 0.0% to 33.7%, with the lowest CV associated with between-season max speed (0.0%) and the highest CV with within-player player HSRD (33.7%). Overall, all sources of variability were higher for HSRD (11.6% to 33.7%), except for between-match variability, which was higher for acceleration distance (30.5%), when compared to the remaining external load metrics. Between-match (for TD, m/min, distance HMLD, accelerations distance), within-player (for HSRD and max speed), and between-role (deceleration distance) presented higher CVs, compared to the other sources of variability.

**TABLE 2:** Variability of full match external loads expressed in raw units and coefficients of variation (%).

	Variability					

	Metric	Between-match	Between-season	Between-position	Between-player	Within-player
SD (90% CI)^a^	TD (m)	313.6 (231.7, 384.7)	102.6 (0.0, 298.7)	268.2 (0.0, 500.2)	418.6 (277.9, 550.1)	337.7 (305.9, 371.5)
	m/min (m)	3.5 (2.8, 4.2)	1.1 (0.0, 2.4)	3.0 (0.0, 5.0)	4.6 (3.4, 6.0)	3.8 (3.5, 4.1)
	HSRD (m)	39.9 (28.4, 50.0)	23.5 (0.0, 49.2)	67.0 (19.3, 105.0)	61.8 (42.9, 79.0)	74.4 (68.4, 79.9)
	HMLD (m)	139.7 (109.9, 170.7)	51.4 (0.0, 119.1)	95.7 (0.0, 171.3)	240.2 (175.2, 298.5)	189.0 (174.0, 202.9)
	Max Speed (km/h)	0.3 (0.0, 0.5)	0.0 (0.0, 0.3)	0.8 (0.0, 1.1)	0.8 (0.0, 1.0)	1.3 (0.0, 1.4)
	Accelerations (m)	1.4 (0.0, 2.7)	0.38 (0.0, 1.9)	9.5 (2.5, 15.6)	10.2 (7.5, 12.9)	9.4 (8.7, 10.0)
	Decelerations (m)	4.6 (0.6, 6.4)	0.0 (0.0, 3.3)	16.3 (3.6, 25.2)	15.9 (11.5, 20.1)	15.4 (14.1, 16.6)

CV (90% CI)^b^	TD (%)	4.6 (3.3, 5.8)	1.1 (0.0, 2.4)	2.9 (0.0, 4.7)	3.5 (2.8, 4.1)	3.8 (3.5, 4.0)
	m/min (%)	4.6 (3.3, 5.79	1.1 (0.0, 2.8)	2.9 (0.0, 5.1)	3.5 (2.7, 4.2)	3.8 (3.5, 4.1)
	HSRD (%)	20.6 (12.9, 28.1)	11.6 (0.0, 24.7)	28.6 (8.0, 47.9)	18.4 (13.4, 23.3)	33.7 (30.7, 36.7)
	HMLD (%)	12.2 (8.8, 15.4)	3.1 (0.0, 6.5)	5.1 (0.0, 9.2)	7.7 (6.0, 9.5)	10.0 (9.3, 10.8)
	Max Speed (%)	3.1 (1.9, 4.1)	0.0 (0.0, 0.9)	2.6 (0.0, 4.3)	1.1 (0.0, 1.7)	5.0 (4.6, 5.4)
	Accelerations (%)	30.5 (21.8, 39.0)	0.0 (0.0, 5.7)	22.6 (3.1, 39.0)	4.5 (0.0, 8.2)	27.3 (24.8, 29.1)
	Decelerations (%)	22.4 (15.8, 29.6)	0.0 (0.0, 5.1)	23.1 (3.3, 37.3)	6.8 (2.8, 9.5)	22.6 (20.8, 24.5)

SD = Standard deviation; CI = Confidence Intervals; CV = Coefficient of variation; HSRD = high-speed running distance; HMLD = high metabolic load distance.

a Values presented in the metric unit of measurement.

b Values presented as a percentage of the mean.

The observed match-to-match variability (combined between-match and within-player) and reference values for interpreting individual changes are presented in Table 3. Based on the model used to identify significant changes (see methods section), between-match individual changes > ± 12% (α = 0.10) and ± 15% (α = 0.05) in full match metrics of TD, m/min, and max speed would be considered unusual and suggest practical significance. For HSRD (83%; 104%), HMLD (32%; 40%), accelerations distance (84%; 106%), and decelerations distance (65%; 82%) these thresholds (α = 0.10; α = 0.05; respectively) are considerably higher.

**TABLE 3:** Reference values for the interpretation of individual changes in match physical performance.

Metric	Observed match-to-match variability (CV 90% CI)^a^	± Limits of agreement (%)^c^		Change (±) required to be practically significant (%)^c^	

		80%	90%	α = 0.10	α = 0.05
TD	5.9 (5.0, 6.9)	10.8	13.8	11.8	14.5
m/min	6.0 (5.0, 7.0)	10.8	13.8	11.8	14.9
HSRD	41.3 (36.1, 46.6)	74.8	95.9	82.7	103.9
HMLD	16.2 (13.5, 19.3)	29.3	37.6	31.8	40.1
Max Speed	5.9 (5.3, 6.5)	10.8	13.8	11.8	14.9
Accelerations	43.2 (36.5, 52.4)	78.4	100.6	83.9	106.1
Decelerations	33.3 (28.3, 38.7)	60.3	77.4	65.1	82.2

CV = Coefficient of variation; TD = total distance; HSRD = high-speed running distance; HMLD = high metabolic load distance.

a Values presented as a percentage of the mean.

b Based on the combined between-match and within-player variability.

c Based on the observed match-to-match variability

### Season trends

**FIG. 1:**
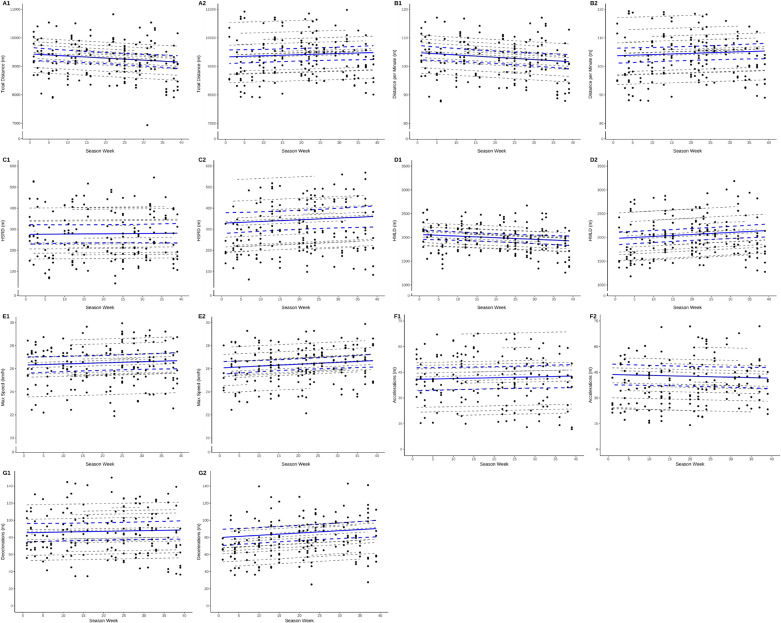
Seasonal trends in each external match load. Numbers represent the season numbers (1 = 2021/2022; 2 = 2022/2023). Total distance (A), m/min (B), HSRD (C), HMLD (D), max speed (E), acceleration distance (F), and deceleration distance (G). Data are presented as individual match observations (black points), individual linear trends (dotted lines) and the overall mean trend (blue bold line) with 90% confidence intervals (blue dotted line). HSRD = high-speed running distance; HMLD = high metabolic load distance.

External match loads throughout the weeks of the season, for each season, are presented in Figure 1. For season 1, the magnitude and directions of the external loads were inconclusive: TD (mean slope: -266.2 m [90% CI: -680.7, 148.7), m/min (-2.9 m [-7.6, 1.7]), HSRD (4.5 m [-50.5, 59.6]), HMLD (-129.2 m [-317.9, 59.5]), max speed (0.4 km/h [-0.2, 1.0]), acceleration distance (1.7 m [-3.3, 6.5]), and deceleration distance (1.5 m [-7.6, 10.6]).

For season 2, there was a seasonal increase in maximal speed attained during matches (0.7 km/h [0.2, 1.3]) and deceleration distance (10.2 m [2.1, 18.5]) throughout season 2. The magnitude and directions of the remaining external load metrics were inconclusive: TD (135.2 m [-173.4, 445.6), m/min (1.5 m [-1.9, 4.9]), HSRD (26.7 m [-26.5, 80.6]), HMLD (164.9 m [17.8, 313.2]), and acceleration distance (-1.9 m [-6.2, 2.2]).

## DISCUSSION

This study explored and described the match-to-match variability of physical demands over a 2-season period in highly trained female soccer players. Moreover, we applied a probabilistic method to monitor individual changes in highly trained female soccer players. Our main findings were that, after considering seasonal trends and inter-position variability, between-match individual changes > ± ~14% in TD, distance per min, and max speed can be considered practically significant in this cohort. Metrics such as HSRD, HMLD, and acceleration and deceleration distance, because of their high variability, presented considerably higher thresholds for relevant changes (> ± 30%).

Overall, absolute match external loads reported from the two seasons analyzed in our study are similar to those reported in other studies and can, therefore, be considered representative of highly trained female soccer players. Recent benchmark studies from international competitions such as the FIFA Women’s World Cup provide valuable context for interpreting match performance standards. For example, Bradley [11] reported that outfield players at the World Cup covered approximately 9,200–10,000 m of total distance per match, with high-speed running distances ranging from 650 to 900 m, depending on playing position. In our sample, total distance values (~9,000 m) and high-speed running distances (~600–700 m, using a similar threshold) fall within this range. This alignment suggests that the physical demands of the Italian Serie A Women’s League, at least for the team analyzed, approach the standards observed at the elite international level. However, some positional and contextual differences remain, particularly in sprint frequency and peak velocities, which may reflect tactical, technical, or stylistic distinctions between leagues. Importantly to note, it is difficult to compare studies given the different criteria used to define intensity zones’ thresholds [3]. For example, Trewin et al. [27] defined HSRD as distance covered > 16.2 km/h, reporting an average of 930 m covered per match. With thresholds similar to our study, Datson et al. [28] reported ~608 m of HSRD per match, while Hewitt et al. [29], and Mara et al. [30] reported 338 and 615 m, respectively. However, both papers defined this cutoff as a ‘sprint’ threshold. Nevertheless, it is reasonable to conclude that even using the same threshold, large discrepancies exist between studies regarding the results and, while Datson et al. [28] and Hewitt et al. [29] analyzed international level players, Mara et al. [30] recruited national level players. When comparing seasons, a significant increase in HSRD from season 1 to season 2 was noted, even though the total distance covered remained similar. This could indicate that players covered a larger portion of their total distance > 19.8 km/h. However, it is important to note that, as reported in our study and others [9, 10, 18, 21, 22], HSRD is highly variable.

Regarding seasonal trends, most of the variables analyzed revealed unclear trends throughout the weeks in both seasons. There was, however, an increase throughout season 2 in maximal speed and deceleration distance. Oliva-Lozano et al. [22] reported increases in HSRD and the number of accelerations throughout the season and a decrease in distance covered. While the distance covered in our study seemed to remain steady throughout both seasons, Oliva-Lozano et al., (2021) reported lower TD at the start of the season and an increase as the season progressed. However, several factors (e.g., team playing style, improved fitness, number of matches, and the competitive context) may explain the different trends in each study. In our study, the team was monitored for two seasons and, even though the technical staff and their fundamental ‘ideas’ remained the same during both seasons, most of the roster was changed from one season to the other. Regarding the playing style, a team that relies strongly on fast transitions and direct play will most likely show higher HSRD values and, possibly, increases in HSRD throughout the season. On the other hand, mid-table teams, such as the team analyzed in the current study, often show a low percentage of ball possession, which is associated with higher TD values. As such, seasonal trends should be analyzed and interpreted from a context-specific perspective, so that practitioners can draw potential practical applications from this type of analysis. However, assessing and monitoring changes in maximal speed through GPS devices may have some limitations. It seems that GPS devices overestimate max speed by 0.11 m/s^−1^ compared to a radar [31].

We decomposed the variability into five components (between-match, between-player, between-position, between-season, and within-player), following a similar approach to Baptista et al. [21] and Oliva-Lozano et al. [22]. Our results align with previous ones since all sources of variability were higher for high-intensity actions (HSRD and acceleration distance [22.4-33.7%]). It has been shown that variability tends to increase with running/action intensity, with match-to-match variability of TD being around 3.1% [32], 2.4% [33], and 5.3% [34] and the HSRD of the respective studies around 9.2, 14.4, and 53%. However, compared to these studies, we could partition the usual sources of variability into their specific components and analyze the elements occurring at the match and player levels by partitioning the observed match-to-match variability into between-match and within-player variability. Results for TD were relatively stable (5% vs. 4%) and similar to Oliva-Lozano et al. [22] (4% vs. 4%) and Baptista et al. [21] (4% vs. 3%). Max speed variability was higher for within-player than between-match (3% vs. 5%), and a similar pattern was reported by Oliva-Lozano et al. [22] (2% vs. 5%) and Baptista et al. [21] (1% vs. 4%). A recent multi-club study in female soccer also reported similar results, specifically in higher speed metrics [23]. Altogether, this suggests HSRD and sprint distances tend to be inconsistent from match-to-match and may need to be considered and informed when tailoring post-match recovery and complementary training. The high match-to-match variability observed in high-speed metrics suggests that large fluctuations in these values are not necessarily indicative of altered fitness or readiness. Therefore, using these variables as primary triggers for adjusting training load or recovery protocols may lead to overinterpretation. Instead, practitioners may benefit from focusing on more stable indicators such as total distance or m/min for day-to-day monitoring. When deviations in high-intensity outputs are observed, they should be interpreted within a broader context that includes tactical roles, opposition quality, player status, and internal load measures. This individualized approach ensures that recovery and training interventions are aligned with meaningful changes rather than normal day-to-day variability. Of course, individual physical characteristics may also contribute to match-to-match variability. Savolainen et al. [35] showed that anthropometric and fitness profiles can influence external load metrics, highlighting the need for individualized interpretation.

On the other hand, acceleration and deceleration variability in our study was much higher than in similar studies, except Myhill et al. [23]. One of those studies used total accelerations (count), and the other the combined total accelerations and decelerations, while in our study we used the accelerations and decelerations distance. Interestingly, Myhill et al. [23] also reported the distance while using an even higher threshold (> 3 ms^2^), which could explain the very high within-player and between-match variability. Additionally, as with HSRD, match exposure to maximum or near maximum efforts is influenced by factors such as opposition level, seasonal variations, and positional differences [36, 37]. Therefore, a high variability of acceleration and deceleration distance is not considered an ‘abnormal’ conclusion, considering similar studies [23].

Lastly, one of the aims of our work was to use the variability data to aid information to the monitoring process and interpret between-match changes in female soccer. The reference values to analyze individual changes in match physical performance are presented in Table 3 and were obtained by a combination of between-match and within-player variability, resulting in 80% and 90% LoA, which were then complemented with thresholds to provide practical significance values (Methods section). This approach is similar to the principles of minimum detectable change and can be used to determine those that are beyond the observed match-to-match variability (negatively or positively). For example, using the values reported in our study, a change of > ± 12% in match TD could be interpreted as higher than usual, using an alpha of 0.1 (90% LoA). Moreover, considering the high variability in high-intensity metrics, it seems that TD or lower intensity metrics may be more suitable to track or detect between-match changes. Additionally, giving context to the changes and understanding the meaningfulness and practical significance of match physical performance variability may help coaches and support staff during the training load management in this context. For example, a marked decrease in HSRD from one match to another may not necessarily mean a poorer performance by the player or a lower physical condition. Similarly, a marked increase in TD might not be associated with physical fitness improvement or a better match performance. Perhaps the player was out of position for the whole match or was constantly overwhelmed by opposition players without teammates covering, for instance. Other contextual match factors can also influence these outcomes. For example, teams that are trailing may engage in more aggressive pressing or transitions, increasing HSRD and acceleration loads. Similarly, playing away from home or under unfavorable scorelines may alter the tactical demands placed on players. Although these factors were not directly modeled in our study, they likely explain part of the residual variability and should be considered in future research and applied monitoring.

### Limitations and Future Directions

Despite our study being a 2-season analysis, the fact that only one team/club was analyzed is a limitation. One of the suggestions made by Oliva-Lozano et al., (2021) was for future studies to include multiple teams, to increase data heterogeneity and possibly decrease the risk of bias. Nevertheless, including two seasons of the same team can be considered a strength of the study, which meant that the sample size was relatively large (n = 27; 344 individual observations). Furthermore, even though practitioners may take the values presented in the study for future reference, the context and competitive level are always important factors to take into consideration. Additionally, only considering the players’ starting positioning without accounting for possible changes during the match can be considered a limitation of the study. While a standardized approach has not been established, it must be acknowledged that players may switch positions throughout the match and our analysis may fail to detect such changes. Thus, future studies should include multiple teams, leagues, and longer periods to increase generalizability and sample diversity. Additionally, integrating contextual variables (i.e., scoreline, match location) and internal load markers may offer a more comprehensive understanding of the factors influencing performance variability.

## CONCLUSIONS

In conclusion, physical performance remains stable across the season and even between seasons, except for HSRD and an increase in deceleration distance covered. However, high-intensity metrics such as HSRD, HMLD, and acceleration and deceleration distance present high variability and, consequently, considerably higher minimum detectable change thresholds. Nonetheless, practitioners must consider that this variability is part of the team sports nature and that many factors can contribute to a decrease or increase from match to match. Thus, training prescription should be varied in both stimulus and intensity and not based on specific benchmarks alone. In this cohort, practically meaningful changes were identified as ± 14% in TD, m/min, and max speed, and > 30% in HSRD, HMLD, and acceleration/deceleration metrics.

## Data Availability

The datasets used and/or analyzed during the current study are available from the corresponding author upon reasonable request.

## Code Availability

The code used in the statistical analysis process of the current study is available from the corresponding author upon reasonable request.
